# Person-centred care to prevent hospitalisations – a focus group study addressing the views of healthcare providers

**DOI:** 10.1186/s12913-022-08198-6

**Published:** 2022-06-20

**Authors:** Cecilie Nørby Lyhne, Merete Bjerrum, Marianne Johansson Jørgensen

**Affiliations:** 1 Research Unit for Nursing and Healthcare, Department of Public Health, Aarhus University, Bartholins Allé 2, 3, 8000 Aarhus C, Denmark; 2 Research Unit, Horsens Regional Hospital, Central Denmark Region, Sundvej 30X, 8700 Horsens, Denmark; 3 Center for Clinical Guidelines, Department of Clinical Medicine, Aalborg University, Soendre Skovvej 15, 9000 Aalborg, Denmark

**Keywords:** Quality improvement, Primary healthcare services, Prevention, Care coordination, Care continuity, Preventable hospitalisation, Person-centered care, Care pathways, Qualitative research, Qualitative interview

## Abstract

**Background:**

The primary healthcare sector comprises various health services, including disease prevention at local level. Research shows that targeted primary healthcare services can prevent the development of acute complications and ultimately reduce the risk of hospitalisations. While interdisciplinary collaboration has been suggested as a means to improve the quality and responsiveness of personal care needs in preventive services, effective implementation remains a challenge. To improve the quality and responsiveness of primary healthcare and to develop initiatives to support the interdisciplinary collaboration in preventive services, there is a need to investigate the views of primary healthcare providers. The aim of this study was to investigate perceptions of preventive care among primary healthcare providers by examining their views on what constitutes a need for hospitalisation, and which strategies are found useful to prevent hospitalisation. Further, to explain how interdisciplinary collaboration can be supported with a view to providing person-centred care.

**Methods:**

Five focus group interviews were conducted with 27 healthcare providers, including general practitioners, social and healthcare assistants, occupational therapists, physiotherapists, home care nurses, specialist nurses and acute care nurses. Interviews were transcribed, and analysed with qualitative content analysis.

**Results:**

Three categories emerged from the analysis: 1) Mental and social conditions influence physical functioning and hospitalisation need, 2) Well-established primary healthcare services are important to provide person-centred care through interdisciplinary collaboration and 3) Interdisciplinary collaboration in primary healthcare services is predominantly focussed on handling acute physical conditions. These describe that the healthcare providers are attentive towards the influence of mental, social and physical conditions on the risk of hospitalisation, entailing a focus on person-centred care. Nevertheless, in the preventive services, interdisciplinary collaboration focusses primarily on handling acute physical conditions, which constitutes a barrier for interdisciplinary collaboration.

**Conclusions:**

By focusing on the whole person, it could be possible to provide more person-centred care through interdisciplinary collaboration and ultimately to prevent some hospitalisations. Stakeholders at all levels should be informed about the relevance of considering mental, social and physical conditions to improve the quality and responsiveness of primary healthcare services and to develop initiatives to support interdisciplinary collaboration.

**Supplementary Information:**

The online version contains supplementary material available at 10.1186/s12913-022-08198-6.

## Background

The primary healthcare sector covers a number of health services, including disease prevention and health promotion at local level [1–5]. Primary healthcare is defined as the first level of contact for the population with the healthcare system, bringing healthcare close to where people live [5]. In modern healthcare, primary healthcare goes beyond the services provided solely by the general practitioner (GP), as it also encompasses other healthcare providers such as nurses, physiotherapists and other care workers [5]. The Organization of Economic Co-operation and Development (OECD) states that good quality primary healthcare may improve health system responsiveness, make healthcare more person-centred and improve population health outcomes, placing a particular focus on services targeting the ageing population and the increasing prevalence of chronic diseases [5].

Research shows that targeted primary healthcare services can prevent the risk of developing acute complications and thus ultimately may reduce the risk of hospitalisations [2, 6]. Such targeted preventive services may include, for example, multidisciplinary diabetes services [7] and assigning a specific GP to nursing homes [8], which have been shown effective to prevent hospitalisations.

Several studies have shown effects of continuity of care, where a history of interaction with the same GP may prevent hospitalisations among older people and among people with chronic disorders, such as diabetes [9–11]. Further, guidelines for integrated people-centred health services highlight how continuity of care could serve as a driver for coordination of care [12]. This has been supported by a study reporting on the preferences of both older people and their relatives [13]. Nevertheless, in preventive services, care continuity and coordination are challenged by confusion and conflicts regarding the roles and responsibilities of the interdisciplinary healthcare providers, specifically when several providers are involved [14, 15].

Preventive services posit organisational support for ‘interdisciplinary collaboration’, defined as efforts to integrate and translate themes and schemes shared by several professions [16]. Further, ‘person-centred care’, defined as care taking a biopsychosocial perspective on health that sees disease as a combined medical, psychological and social problem, could facilitate preventive services, as it emphasises the personal preferences, needs and values [17, 18]. While interdisciplinary collaboration in preventive services has been suggested as a means to improve the quality and responsiveness of personal care needs, effective implementation remains a challenge [19–21]. Only few studies have provided insight into the collaborative dynamics among primary healthcare providers and addressed their perceptions on what constitutes preventive care work and how individual care needs are met. The knowledge is however important in the effort of improving the quality and responsiveness of primary healthcare and the development of initiatives that may support interdisciplinary collaboration in the preventive services [5].

The aim of this study was to investigate perceptions of preventive care among primary healthcare providers by examining their views on what constitutes a need for hospitalisation, and which strategies are found useful to prevent hospitalisation. Further, to explain how interdisciplinary collaboration can be supported with a view to providing person-centred care.

## Methods

### Design

This study was designed as a qualitative study based on qualitative inductive content analysis methodology [22–27]. Focus group interviews were applied to capture the perceptions of preventive care among primary healthcare providers, including their views on collaborative encounters across municipalities and general practices, in the endeavours to provide person-centred care [28, 29].

### Setting

The study was conducted in four municipalities in the catchment area of one regional hospital in the Central Denmark Region. The Danish healthcare system is tax-funded and based on open access in line with e.g. other Nordic countries and Australia [30, 31]. As part of the primary healthcare services, the municipalities are responsible for prevention and rehabilitation, including practical and nursing assistance in accordance with the Danish Health Care Act [31] and the Social Service Act [32]. In the preventive services aiming to prevent acute complications among older people and people with chronic disorders, collaboration between municipal healthcare providers and the GPs is essential. The GPs have medical authority, can refer people to welfare services, such as home care and nursing assistance, and are gatekeepers to specialised healthcare, including hospital referral [31, 33].

### Informants

General practitioners, municipal managers and nurse consultants in four municipalities met with healthcare administers and specialist nurses at the regional hospital, and CNL identified healthcare providers with a key role in the preventive services. Municipal leaders supported the project and allowed municipal healthcare providers to participate in focus group interviews during working hours. Healthcare providers fulfilling the inclusion criteria (Table 1) were invited to participate by email from local municipal consultants and CNL based on purposive sampling [34]. None refused to participate in the interviews.

**Table 1:** Inclusion criteria

Occupation	GP; social and healthcare assistant; acute care nurse manager; homecare nurse; nurse with work experience from a nursing home; nurse with work experience with preventive services in home settings; nurse with experience in intermediate care (e.g. municipal acute care beds); healthcare provider with experience from rehabilitation teams or therapist with experience in municipal visitation
Work function	Involved in the preventive services in the primary healthcare sector in one of the four included municipalities in the Central Denmark Region

We included 27 primary healthcare providers (Table 2). The participants performed key work functions and care tasks in the preventive services in the four included municipalities with 20,000–60,000 inhabitants. The participants served for similar groups of people, but they had different professional backgrounds and work functions in the preventive services. Thereby, the focus groups provided authentic insights into interdisciplinary collaboration through the direct access to social interaction dynamics.

**Table 2:** Main characteristics of participants

Number of participants	Occupation	Work setting	Key work functions and care tasks
7	Registered nurse (RN)	Healthcare setting, municipality	Conducts primary care tasks (e.g. intermediate care facilities); visitation (i.e. assessing and referring people to social and healthcare services provided by the municipality)
4	General practitioner (GP)	General practice	Serves as a medical doctor with special interest in interdisciplinary and cross-sectoral collaboration
4	Nurse manager (NM)	Healthcare setting, municipality	Conducts management tasks related to preventive services (e.g. intermediate care facilities, nursing home)
4	Social and healthcare assistant (SHA)	Homecare, municipality	Performs practical and care tasks in home setting for people eligible for healthcare and homecare
3	Acute care nurse (ACN)	Acute care in home setting, municipality	Serves as a specialised municipality staff nurse conducting acute care tasks in home setting
2	Occupational therapist (OT)	Rehabilitation service, municipality	Provides rehabilitation services to people referred from the municipality visitation
2	Homecare nurse (HN)	Home nursing, municipality	Provides homecare, including health and medical care
1	Physiotherapist (PT)	Rehabilitation service, municipality	Provides rehabilitation services for people with functional impairments

### Data collection

Five focus group interviews were conducted between 1 September and 31 December 2019. The focus groups consisted of four to nine informants; each focus group comprised municipal healthcare providers and one or two GPs. In the fifth interview, selected participants from the previous four interviews were invited to elaborate further on aspects mentioned in the earlier interviews; this interview focused particularly on the interdisciplinary communication and coordination across care settings.

All focus group interviews were conducted by CNL, a public health researcher experienced in conducting qualitative interviews. MJJ, a senior researcher with expertise within healthcare services research and clinical experience from healthcare practice, observed the focus group interviews to ensure that aspects of relevance to the aim of the study were covered. Each focus group interview lasted approximately 1.5 h. The interviews were conducted in meeting rooms at the premises of the included municipalities without disturbance from colleagues or managers, and only participants were present.

The semi-structured interview guide comprised four predefined themes: avoidable hospitalisations, collaboration, care pathways and distribution of responsibility. In addition, one open theme was included to give the participants the opportunity to discuss matters of relevance that were not addressed through the predefined themes [see Additional file 1]. The interview guide was based on issues from the background literature, meetings and informal discussions with health service researchers, GPs and healthcare providers and managers in municipal healthcare and hospital settings to ensure that relevant questions and explanatory assumptions were considered.

CNL encouraged group dynamics by using different types of questions [see Additional file 2] [35]. Direct questions structured the interview and facilitated further discussions. Specifying questions invited further elaboration on implicit aspects. Interpreting questions ensured valid understanding of aspects presented by participants. Follow-up questions encouraged discussion of an aspect mentioned by a participant. Finally, probing questions were used to further explore the healthcare providers’ perceptions and experiences by asking for examples and descriptions of concrete episodes.

All interviews were audiotaped. After each interview, immediate impressions and considerations were noted in a logbook. Interviews were concurrently discussed with MJJ to strengthen the reliability of the data collection.

### Data analysis

Interviews were transcribed verbatim, including pauses, laughter and changes in breathing, tone of voice or vocal pitch [35]. The transcripts were analysed using inductive content analysis [22–26, 36], with a focus on how the manifest and latent content describes and explains the perceptions and experiences of healthcare providers serving person-centred care though interdisciplinary collaboration. The analytical process focused on the healthcare providers’ perceptions and experiences and on the group dynamics. Discussions reflecting diverse perceptions and consensus among the healthcare providers were examined.

The inductive content analysis was performed in four steps. First, CNL read the transcripts to obtain an overall impression of the interviews and noted initial analytical considerations in a logbook. Second, to ensure that the analytical focus was directed towards the study aim, meaning units from all interviews were systematically extracted through the use of two analytical questions: a) What constitutes a need for hospitalisation from the perspectives of primary healthcare providers? b) Which preventive services are useful to prevent hospitalisations from the perspectives of primary healthcare providers? In the initial coding process, CNL and MJJ independently coded selected sections from the transcripts using the analytical questions. The two coders complemented each other professionally and methodologically as CNL is experienced in qualitative content analysis methodology within the field of public health, including health promotion and disease prevention, whereas MJJ has expertise within healthcare services research and clinical experience from healthcare practice. Thus, the two coders were able to capture a wide range of aspects related to prevention, clinical healthcare, and healthcare service organisation. After the initial coding, CNL and MJJ discussed coding decisions to clarify and reach consensus on the meaning of the analytical questions to ensure consistency in the meaning units extracted. The inter-coder agreement was 89% [26]. Discrepancy was found for meaning units on hospital discharge and readmissions, and consensus was reached that these aspects were not considered relevant, as the focus of the study was the primary healthcare services for the prevention of acute complications and ultimately hospitalisation. After this clarification, CNL coded all interviews. Third, all extracted meaning units were analysed, focusing on resemblance in meaning, and organized into three descriptive categories. During categorisation, the logbook and the non-verbal communication features in the interview transcripts such as laughter, changes in breathing, tone or pitch was used to situate the meaning units in the context by capturing the latent content. Forth, the categorised meaning units were compared to identify meaningful patterns, and one overarching explanatory theme emerged [23, 37].

To strengthen the validity and reliability of the meaning units, the categories and the overarching theme, the analytical process was conducted in continuous dialogue among CNL, MJJ and MB. MB is experienced in content analysis methodology in the fields of public health research and rehabilitation. Finally, as the interviews were conducted in Danish, key quotations from the interviews were identified and translated into English. First, the quotes were translated by CNL to ensure consideration of the context. Second, the draft translations were revised and qualified by MJJ and a translator, and the final versions of the key quotations were incorporated into the manuscript.

## Results

**Fig. 1:**
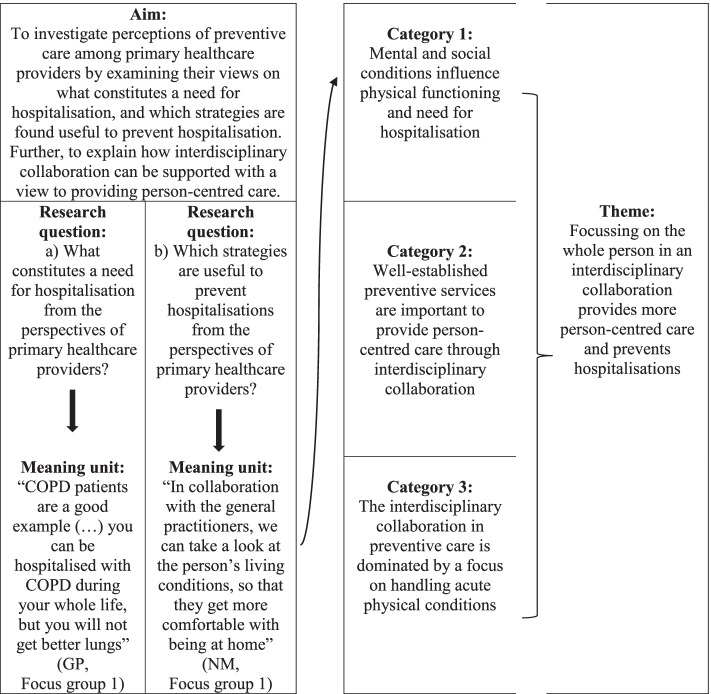
The analytical process, exemplified for category 1

The analytical process (Fig. 1) revealed three categories describing what primary healthcare providers perceive as preventive healthcare. These categories were disclosed by examining their views on what constitutes a need for hospitalisation, and which strategies they found useful to prevent hospitalisation. The three categories were: 1) mental and social conditions influence physical functioning and need for hospitalisation, 2) well-established preventive services are important to provide person-centred care through interdisciplinary collaboration and 3) the interdisciplinary collaboration in preventive care is dominated by a focus on handling acute physical conditions.

### Category 1: Mental and social conditions influence physical functioning and need for hospitalisation

The preventive services were primarily oriented towards physical conditions, especially among older people and people with chronic diseases. Nevertheless, the healthcare providers were attentive to the influence of mental, social and physical conditions on health outcomes, functioning and, ultimately, risk of hospitalisation.

The preventive services are mainly focussed on reducing existing complications by dealing with acute physical conditions, such as chronic obstructive pulmonary disease (COPD) exacerbation or glycosuria (glucose in urine), which challenged the provision of relevant and timely preventive services aligned with the individual person’s care needs. However, the healthcare providers emphasised the dynamics between mental, social and physical conditions, as important elements influencing health state and disease development. Both GPs and municipal care providers were aware of the importance of considering the person’s individual care needs to prevent the development of conditions that require hospitalisation. The importance of considering the individual person’s life situation in relation to the physical disorder was highlighted, as the home environment could be an important contributing factor for a chronic disorder to develop into an acute condition, which may carry a risk of hospitalisation. For example, living on the second floor while having COPD could lead to hospitalisation, which might have been prevented.

“We had someone who lived on the second floor in a building with no lift, and he had COPD, and after arriving back home and climbing the stairs, he was ready to be hospitalised” (PT, Focus group 3)

The provision of care in the person’s home, nursing homes or acute care beds in the municipal-based health centres was perceived as a unique possibility to assess the person’s needs and to gain a more complete understanding of “the whole person” because such settings allow the healthcare provider to become familiar with the person’s daily routines and functioning. The understanding of “the whole person” was informed by mental, physical and social aspects, including links between mental and physical health, behaviour and mood, social relations, social contact, loneliness and home environment, including assistive devices, technical aids and home adjustments. Especially, the healthcare staff providing care in homely settings stressed the importance of seeing the person instead of seeing a diagnosis. However, frustrations were encountered when none of the available initiatives deal with aspects that are not directly health-related, as the tools and documentation systems that are systematically implemented tend to focus on physical conditions and acute illness.

“… the system doesn’t really support all those underlying reflections. It is more specific and here and now: Have you tested the urine? and… uhm… what else does it [the system] say?” (PT)

“Have you measured blood sugar?” (ACN)

“Yes, […] if the person is diabetic, and always contact a doctor if the score has this [value]. But I just think that there are a great many other reflections about an acute condition” (PT) (Focus group 4)

The healthcare providers’ possibilities for delivering care that considers the whole person and focuses on the individual care needs and preferences are challenged by a pronounced emphasis on physical conditions in the care practices and in the visitation to healthcare services. This was seen as a barrier for providing preventive services customised to the whole person and the individual care needs, as no individual care plan was outlined to reflect the person’s needs for care and personal preferences.

“People are allocated specific services on the basis of their care needs, and a care need for mental support on a daily basis is not included in any of the care packages (SHA).

“No, but you may say that this speaks into what the purpose is of the psychiatric support, and then there needs to be an action plan for this.” (NM, Focus group 4)

### Category 2: Well-established preventive services are important to provide person-centred care through interdisciplinary collaboration

The need for well-established preventive services that align with the person’s care needs was highlighted as a response to the existing preventive services, which are frequently changing and involve different healthcare providers.

Frequent replacements of the preventive services were seen in the municipalities, and new initiatives rarely became well integrated, which challenged the use and the trustworthiness. Additionally, this was often followed by frequent changes in the access points and eligibility criteria, which made it difficult to keep updated on the current preventive services and the access criteria for these services in the municipality.

“But do we still have that service available?” (HN)

“I don’t think so” (RN)

“Me neither” (NM)

“It has certainly not been used for a long time“ (RN) (Focus group 4).

GPs were often inclined to refer patients to the municipal health services that they were familiar with, e.g. the municipal acute care beds, which offer short-term stays for people with constant care needs. Nevertheless, there was a need for other preventive services that match the care needs of the persons living in the municipalities and for establishing more stable and fixed preventive services.

“If I have someone [with severe care needs], but all the municipal acute care beds are taken, then the only solution would be to hospitalise” (GP) (Focus group 5).

Preventive services and actions were considered more meaningful when they met the person’s needs rather than patient outcomes (e.g. avoidable hospitalisations), which primarily had organisational value. Especially, the nurses and GPs underlined the adherence to own professional norms and values as important in relation to the incentives given by the healthcare organisation to reduce hospitalisations. Concerns were raised that people who felt unsafe in their own home, and therefore preferred to be hospitalised, were difficult to accommodate within the existing preventive services.

“None of us should go home from work feeling sad because we admitted a person to the hospital who didn’t need it. But sometimes you may think so, but that’s not how it should be. Still, it can be shitty having to admit someone. Actually, I can go home sad after work if I have hospitalised someone because that was the only option.” (GP) (Focus group 2)

Providing care for the same person and having repeated visits in the person’s home was seen as a valuable possibility for assessing the person’s general health state and well-being, living conditions, home environment and social relations. In this way, changes in health state could be detected, which made it possible to refer people to relevant and timely services. Still, frequent shifts in the municipal healthcare staff and changing work schedules were barriers for referring people to services that corresponded to the person’s needs. Further, the time for delivering care for each person was restricted, regardless of the person’s needs. Lack of care continuity and coordination among the involved healthcare providers may cause unfavourable confusion and uncertainty, especially among older people with extensive care needs and among people with chronic conditions, who are already in a vulnerable situation with limited health functioning and uncertain future health state.

“I often visit people who say, ‘oh, first comes one provider and then someone else; one asks one thing, and another answers something else’, and… well… they may get really confused, and that does not promote the feeling of safety that should come from ‘having someone in control of my situation’” (HN) (Focus group 3)

### Category 3: The interdisciplinary collaboration in preventive services is dominated by a focus on handling acute physical conditions

A focus on handling acute physical conditions dominated the preventive services. This restricted the healthcare providers’ opportunities for preventing acute complications from arising through interdisciplinary collaboration.

In the preventive services, homecare providers were seen as a starting point for the initiation of early and timely provision of services to people at risk of hospitalisation. The healthcare providers adhered to guidelines for handling acute situations through interdisciplinary collaboration. The homecare providers may call the acute care nurse when observing initial changes in the person’s health state. Then the acute care nurse can follow up and contact the GP if considered relevant. In this way, the responsibility for observing persons in need of care and for delivering this information to a municipal nurse remains with the homecare providers. Clear communication between homecare providers and acute care nurses was perceived a key matter to initiate preventive services that aligned with the individual person’s needs in due time. The use of uniform terminology that translated healthcare providers’ observations to simple messages, such as labelling the person’s health state as red, yellow or green, helped clarify how to respond to a person’s actual needs and initiate relevant actions accordingly. Especially, communication and sharing of information were perceived as key to initiate preventive services. Therefore, educational information and training of healthcare assistants were perceived important.

“I think that it’s all about continuous updating of new employees and keeping up with the geriatric knowledge on what it is important to look for, and often also giving them the comfort of calling us [the acute care team] and entering into a dialogue with them [to make them aware] that yes, I will ask them about something when they call me, because that sometimes comes as a surprise to some” (ACN) (Focus group 2)

Awareness of other healthcare providers’ skills and competencies were perceived as prerequisites for the provision of person-centred care through interdisciplinary collaboration. The interdisciplinary collaboration was challenged when healthcare providers primarily focussed on the execution of their own work tasks, whereas the interdisciplinary collaboration was successful when healthcare providers had confidence in each other’s competencies and were trained to work in a team with a clearly outlined common goal. Person-centred care required clearly defined work functions for each of the healthcare providers in the teamwork, but it also required clear lines of communication among healthcare providers so that targeted care services could be initiated immediately.

“There has been a fixed interdisciplinary team […] Therefore, we have been able to take immediate action since I didn’t have to write in the system first and then wait for the physiotherapist to come out to the patient” (RN) (Focus group 4)

Collaboration between the GPs and the municipal healthcare providers, and internal collaboration between the municipal healthcare providers, was a prerequisite for successful integration of the expertise provided by different healthcare disciplines as they complement each other. Nevertheless, the general practices, the individual municipal institutions, such as nursing homes and acute care beds, and the individual municipality were all focused on their own institutional goals and short-term budgets, and this was reflected in the care work of the healthcare providers. A strong focus on own primary work tasks and competing priorities across institutions tended to draw away the attention from prevention, person-centred care and interdisciplinary collaboration.

”We don’t focus on the task. We focus on our own little box or ‘business’ and that everything should come together for each individual area, and not so much [putting] the patient or the citizen in the centre” (NM)

“The discharge summaries are now delivered with a colour. Green, yellow and red. It has started now. At least in our clinic. We are excited to see what it can do… how the collaboration will be, but also if it works as intended, that we can catch the citizens at risk [of hospitalisation].” (GP) (Focus group 5)

## Discussion

The transversal analysis [23, 37] of the three categories revealed one overarching explanatory theme: ‘Focussing on the whole person in an interdisciplinary collaboration provides more person-centred care and prevents hospitalisations’. The theme explains that the healthcare providers are attentive towards the influence of mental, social and physical conditions on the risk of hospitalisation, which highlights the importance of delivering care that considers the whole person. However, the strong orientation towards the handling of acute physical conditions is a barrier for interdisciplinary collaboration in preventive services. By focusing on the whole person, it might be possible to provide more person-centred care through interdisciplinary collaboration and ultimately to prevent some hospitalisations. In this way, the healthcare providers’ perceptions of what constitutes a need for hospitalisation derives from the understanding that not only the physical condition but also mental and social factors are important ingredients. Thus, this adheres to the biopsychosocial model of health and disease [38]. To grasp the healthcare providers’ perceptions of what constitutes a need for care and hospitalisation, ‘the concept of care’ by Mol et al. [39] can be useful, as care is introduced as a multiple formulation defined according to the specificities of the situation. Additionally, Mol et al. [40] have argued that ‘care in practice’ opposes the systems of control that are pervading the care work in particular. In this way, exerting control of care activities through the proliferation of checks, rules and regulations is a strategy that implies the objectification, centralisation, disembodiment, formalisation and standardisation of work practices. In contrast, the quality of care can be improved through the recognition of the generative nature of care practices. Thus, the present findings of the healthcare providers’ perceptions complement the theory by highlighting the importance of considering the person who need care, not with reference to disease and standardised categories, but through careful consideration of the situation, including the specific person’s care needs.

The findings show that the healthcare providers found that lack of care continuity and coordination caused unfavourable confusion and uncertainty among the people who were already in a vulnerable situation. This is supported by studies examining the preferences of older persons, which highlight the importance of continuity of care to prevent hospitalisation [41, 42]. For example, a trustful relationship with healthcare providers was a motivator for engaging in prevention, and a trustful relation can be established through specific actions, including solving practical issues, such as installing loft insulation in the home [42]. This underscores the importance of providing care that aligns with a biopsychosocial approach [38] and corresponds with the preferences of the person in need of care. In support of these qualitative findings, quantitative studies have shown that interactions between the mental, social and physical conditions influence the need for hospitalisation [43–51]. For example, a cohort study has shown a dose–response relationship between perceived stress and hospitalisations for ambulatory care-sensitive conditions, including both chronic somatic conditions and mental health conditions [50]. This aligns with the concept of care by Mol et al. [39]. Our findings imply that the preventive care work may be adapted to the person and the specificities of the situation. In this way, it might be possible to provide person-centred care that complies with the preferences of the person by drawing on interdisciplinary collaboration between a range of different types of healthcare providers.

In this study, the findings showed that competing priorities across institutions and the healthcare providers’ focus on own work tasks tended to draw the focus away from prevention, person-centred care and interdisciplinary collaboration. In addition, a study that included care providers from primary and secondary healthcare and the social care sector showed that community-based intermediate care services remained under-used and were perceived as somehow separate from the well-integrated healthcare services [15]. Thus, to support the interdisciplinary collaboration between primary healthcare providers to achieve more person-centred care, there is a need for sustainable solutions that bridge the division between the healthcare sector and the social services [12, 42, 52, 53]. Policies that aim to strengthen the integration of social care, acute and primary healthcare could focus on the patient relation and patient safety. In this way, it might be possible to further engage the healthcare managers and to support the care providers in their interdisciplinary collaborations to make better use of the various competencies. Ultimately, this might contribute to the provision of care that accounts not only for the directly derived health-related issues with implications for acute care, but also for the mental and social aspects that greatly influence health state and disease development.

### Methodological considerations

The use of focus group interviews provided insight into the perceptions of collaborative municipal healthcare providers and GPs. A mix of the healthcare providers corresponding to the inclusion criteria participated in the focus groups by which the study results present a true reflection of the interdisciplinary collaborations in the primary healthcare sector focusing on the preventive services. To strengthen the internal validity, we based the aim, the analytical questions, and the interview guide on background literature, informal meetings with key actors from the healthcare sector, and discussions with health service researchers. Thus, the focus group interviews comprised actual and relevant information on preventive services across municipal healthcare settings and general practice.

The richness of the data derived from both the interview transcripts, which reflected healthcare providers’ perceptions, and the logbook, which contributed with descriptions from the interviews, including the physical surroundings, atmosphere and group dynamics during interviews, allowed in-depth analysis by enabling the emergence of the latent content of meaning. The qualitative content analysis was useful, as it allowed the rich amount of data to be organised into categories. The use of analytical questions ensured that data could be coded according to aspects of relevance to the aim, which further strengthened the validity of the findings.

The study is limited by the inclusion of the participants, as only primary healthcare providers participated in the interviews. Therefore, the perspectives of persons with care needs, care providers from the social sector, and clinicians from the hospital are not represented. Still, the primary healthcare providers are important actors in the preventive services, as the municipalities are responsible for the prevention and rehabilitation, and GPs have the medical authority and act as gatekeepers to specialised healthcare, home care and nursing assistance.

Further, in relation to the external validity of the study, the present study is conducted in a tax-funded and open access system. Thus, in transferring the results to other contexts, the organisation of the healthcare system should be considered, including the access points and the funding of healthcare services, as should the roles and responsibilities of the involved institutions and care providers.

### Implications for practice

The findings stress the importance of involving stakeholders at all levels and informing about the relevance of social and mental conditions, as they may influence the general health state and the risk of hospitalisation. This could form the basis for developing targeted strategies in local primary care settings to improve care coordination across disciplines and institutions. Further, targeted strategies to establish continuity in the care work focusing on building a trustful relation between healthcare provider and the person with care needs are warranted in the provision of person-centred care.

To engage and motivate policy-makers, healthcare management and healthcare professionals, the implications for patient safety can be used as an entry point to add legitimacy to developing and integrating preventive services to reduce risk of hospitalisations. The development and sustainable implementation of person-centred care in local primary care settings may be supported by evidence-based practices and co-production trajectories.

### Implications for research

There is a need for studies that examine the collaborations across municipalities, general practices, the social sector and hospitals. Additionally, studies that may deliver recommendations are needed, specifically on how preventive services can be sustainably integrated into the interdisciplinary collaborations that are currently focused on handling acute physical conditions. This includes research that aims to convey the evidence to policy-makers, healthcare management, administers and healthcare professionals in all sectors, as this may support and stimulate co-production and local translation of evidence-based preventive services.

Further, there is a need to gain more knowledge from the perspectives of persons with care needs and to detail their ability and motivation to engage in preventive services, as this will allow us to focus on developing best practice guidance on care continuity and coordination.

## Conclusions

This study showed that interdisciplinary collaboration is challenged when the primary focus in preventive services is directed on the handling of acute physical conditions. Nevertheless, primary healthcare providers are attentive towards the influence of mental, social and physical conditions on the risk of hospitalisation and towards the importance of serving care focussed on the whole person. By focusing on the whole person, it might be possible to provide more person-centred care through interdisciplinary collaboration and, ultimately, to prevent hospitalisations. The findings stress the importance of involving stakeholders at all levels and informing about the relevance of social and mental conditions that may influence the general health state and the need for hospitalisation. Thereby, the findings can contribute to improve the quality and the responsiveness of the primary healthcare services and to develop initiatives that may support the interdisciplinary collaboration in the preventive services.

## Supplementary Information

**Additonal file 1.** Interview guide.

**Additonal file 2.** Types of interview questions.

## Data Availability

The datasets generated and/or analysed during the current study are not publicly available due to confidentiality issues, but they are available from the corresponding author on reasonable request.

## Abbreviations

ACN: Acute care nurse
COPD: Chronic obstructive pulmonary disease
GP: General practitioner
HN: Homecare nurse
NM: Nurse manager
OT: Occupational therapist
PT: Physiotherapist
RN: Registered nurse
SHA: Social and healthcare assistant
