# Supplementary material for: Person-centred care to prevent hospitalisations – a focus group study addressing the views of healthcare providers
Source: BMC Health Serv Res. 2022 Jun 20;22:801. doi: 10.1186/s12913-022-08198-6 (PMC9210672; doi:10.1186/s12913-022-08198-6)
Supplement: Supplementary file 1 — Additonal file 1. Interview guide. [file 12913_2022_8198_MOESM1_ESM.docx]

Additional file 1. Interview guide

| **Themes** | **Interview questions** |
| --- | --- |
| Briefing | Good afternoon, we are happy to see all of you today.  My name is Cecilie, and I am a researcher at Aarhus University. I collaborate closely with Marianne, who is also attending today.  We have invited you to gain insight into your working experiences with avoidable hospitalisations and collaboration with other healthcare providers.  I will introduce some themes that I would like you to discuss, and I may also ask you some questions during your discussions. Marianne is here as an observer. She might take some notes and may also ask questions.  We will start with a presentation round; you may introduce yourself with name and job title. I will audio record the interview.  [*Presentation round*]  Do you have any questions before we begin?  The themes are: avoidable hospitalisations, collaboration, care pathways, distribution of responsibility and a non-defined theme [*five printed coloured A4 cards with the theme labels are presented*], and you will get the opportunity to discuss matters of relevance if you find that some issues have not been addressed through the predefined themes. Now, I will ask you to decide the order in which you would prefer to discuss the themes in your group, and then you can place the cards in that order on the table.  *[Participants decide the order and place the cards at the middle of the table]* |
| Avoidable hospitalisations | Do you have examples of a hospitalisation that could have been prevented?   - What were the challenges? Which healthcare providers were involved? - Do all the people who are admitted to hospital need hospitalisation? - Based on your work experiences, are there any specific disorders that could be better managed in the primary healthcare sector to prevent hospitalisation? |
| Collaboration | Do you have examples of collaborations with other healthcare providers on the care of a specific person?   - Can you describe a situation in which you were in contact with other healthcare providers? - Do you have practical examples of collaborations between municipal healthcare services and general practice? For example, who in the municipality will the GP contact when seeing a person who is not eligible for hospital admission, but who is in need of targeted preventive services to reduce the risk of hospitalisation? - How could the collaboration be strengthened between healthcare providers in the preventive services to provide better care pathways and lower the risk of hospitalisation? |
| Care pathways | Can you give examples of care pathways for people who need special actions or targeted service to prevent hospitalisation?   - Can you describe a recent case or situation that you have experienced? Does this case belong to a group of people with special needs? What are special about these? - Do you have other examples from your work? - Which arrangements would it require from the internal collaboration in the municipalities to ensure targeted care work? Which arrangements would it require from the collaboration between healthcare providers and professionals across municipalities, general practice and hospitals? |
| Distribution of responsibility | What do you see as your primary responsibilities in the care pathways across sectors and in your care work?   - Can you give examples of what you see as other healthcare providers’ responsibilities? - What are your opportunities to initiate preventive services? - When do you depend on other healthcare providers? Examples? - Who has the main responsibility for the individual person and the care pathway? - Who can you contact to get the help or knowledge needed on the opportunities to initiate preventive services? |
| Non-defined theme | Based on your working experiences, are there any matters that you would like to discuss with relevance to avoidable hospitalisations and collaborations, which have not been covered? |
| Debriefing | Now, we have five minutes left. Is there anything that you would like to add or elaborate on? Any further questions? Thank you all for your time. |
