# Supplementary material for: Person-centred care to prevent hospitalisations – a focus group study addressing the views of healthcare providers
Source: BMC Health Serv Res. 2022 Jun 20;22:801. doi: 10.1186/s12913-022-08198-6 (PMC9210672; doi:10.1186/s12913-022-08198-6)
Supplement: Supplementary file 2 — Additonal file 2. Types of interview questions. [file 12913_2022_8198_MOESM2_ESM.docx]

Additional file 2. Types of interview questions

| **Types of interview questions** | **Questioning during interview (examples from focus group interviews illustrated with quotes)** |
| --- | --- |
| Direct questions | *“Can you give an example of a case you thought went well in order to prevent hospitalisation?”* (Focus group 4) |
| Specifying questions | *“For how long can a person stay at the facility with municipal acute care beds? Are there any rules for that?”* (Focus group 3) |
| Interpreting questions | *“Now I need to understand this right. These municipal acute care beds are actually a sound initiative to exactly prevent a hospitalisation for a person like the one you were talking about?”* (Focus group 1) |
| Follow-up questions | *“To further elaborate and based on what you say, then this could offer some opportunities to prevent hospitalisations?”* (Focus group 1) |
| Probing questions | *“So what you are touching upon here is something that is related to the good collaboration?”* (Focus group 5) |
